# Cardiovascular Mortality in Ovarian Cancer Patients: An Analysis of Patient Characteristics Using the SEER Database

**DOI:** 10.3390/medicina59081476

**Published:** 2023-08-17

**Authors:** Ismail Abdulrahman Al-Badawi, Osama Alomar, Saud Owaimer Alsehaimi, Mohammed Ziad Jamjoom, Nadia Ahmed Abdulmalik, Ibtihal Abdulaziz Bukhari, Abdullah Alyousef, Safa Alabdrabalamir, Saeed Baradwan, Ahmad Sayasneh, Saad M. S. Alqarni, Ahmed Abu-Zaid

**Affiliations:** 1 Department of Obstetrics and Gynecology, King Faisal Specialist Hospital and Research Center, Riyadh 11564, Saudi Arabiasss056sss@gmail.com (S.O.A.); 2 Department of Obstetrics and Gynecology, King Fahad Armed Forces Hospital, Jeddah 23311, Saudi Arabia; 3 Department of Obstetrics and Gynecology, College of Medicine, Princess Nourah Bint Abdulrahman University, Riyadh 11671, Saudi Arabia; 4 College of Medicine, Almaarefa University, Riyadh 11597, Saudi Arabia; 5 Riyadh Second Health Cluster, Riyadh 11622, Saudi Arabia; 6 Department of Obstetrics and Gynecology, King Faisal Specialist Hospital and Research Center, Jeddah 23431, Saudi Arabia; 7 Faculty of Life Sciences & Medicine at Guy’s, The School of Life Course Sciences, King’s College London, London WC2R 2LS, UK; ahmad.sayasneh@gstt.nhs.uk; 8 Department of Gynaecological Oncology, Guys’ and St Thomas’s NHS Foundation Trust, London SE1 7EH, UK; 9 Department of Obstetrics and Gynecology, Abha Maternity and Children’s Hospital, Abha 62562, Saudi Arabia; 10 College of Medicine, Alfaisal University, Riyadh 11533, Saudi Arabia

**Keywords:** ovarian cancer, cardiovascular disease, mortality, survival, SEE

## Abstract

*Background and Objectives*: Cardiovascular disease (CVD) is a major contributor to the high mortality rate among individuals with ovarian cancer. Nevertheless, there is limited understanding regarding the specific patient attributes that might impact the risk of CVD in this group. *Materials and Methods*: A retrospective cohort study was performed using the SEER database to analyze primary ovarian cancer cases from 2000 to 2019. Multivariable logistic regression analysis was employed to identify patient characteristics linked to cardiovascular mortality. *Results*: The cohort included 41,930 cases of patients who were alive, 54,829 cases of cancer-related deaths, 3003 cases of cardiovascular-related deaths, and 10,238 cases with other causes of death. Poorly differentiated cancer cells and distant metastasis were associated with a higher risk of cardiovascular mortality. Logistic regression analysis identified age, year of diagnosis, race, laterality, and staging as significant risk factors for cardiovascular cause of death. The risk of cardiovascular cause of death was lower in patients aged 31–60 and higher in those aged over 60 years old, and the risk also increased with a later year of diagnosis. Patients who were not white were at a higher risk of cardiovascular cause of death. Additionally, bilateral ovarian cancer and distant staging disease were linked to elevated risks of cardiovascular cause of death. *Conclusion*: Cardiovascular mortality is a significant concern in ovarian cancer patients, and several patient characteristics are associated with an increased risk. Our study suggests that targeted interventions to improve cardiovascular health in high-risk patients, such as those with comorbidities or an advanced stage at diagnosis, may improve survival in this population.

## 1. Introduction

Ovarian cancer is a difficult disease to manage due to its aggressive nature and high recurrence rates. Despite advancements in diagnosis and treatment, it remains a leading cause of cancer-related deaths in women in the United States. This gynecologic cancer originates in the ovaries and is often diagnosed at an advanced stage. In 2021, around 22,000 women were estimated to be diagnosed with ovarian cancer in the United States, resulting in over 13,000 deaths. The prognosis for ovarian cancer is generally poor, with a five-year survival rate of approximately 48% [1,2]. 

Recent studies have suggested that cardiovascular disease (CVD) may be a major contributor to morbidity and mortality among ovarian cancer patients. This study aims to explore the incidence and patient characteristics associated with cardiovascular mortality in ovarian cancer patients using the Surveillance, Epidemiology, and End Results (SEER) database [3,4,5].

CVD is a prominent global mortality factor, resulting in more than 17 million annual fatalities. It includes a wide range of conditions, such as coronary artery disease, stroke, and heart failure. Although cancer is not typically associated with CVD, recent studies have suggested that cancer patients may be at increased risk of developing CVD due to factors such as cancer treatments, inflammatory responses, and shared risk factors [4,5].

The primary objective of this study is to explore the incidence and patient characteristics associated with cardiovascular mortality in ovarian cancer patients using the SEER database. Specifically, we aim to determine the incidence of cardiovascular mortality among ovarian cancer patients in the SEER database and identify patient characteristics (for example, age, comorbidities, and cancer stage) that are associated with an increased risk of cardiovascular mortality in ovarian cancer patients. By exploring these factors using the SEER database, we hope to gain a better understanding of the incidence and patient characteristics associated with cardiovascular mortality in ovarian cancer patients. Ultimately, this research may inform clinical practice and help identify areas for future research and intervention.

## 2. Materials and Methods

This study aimed to explore the risk factors for cardiovascular cause of death (COD) among ovarian cancer patients using the SEER Research Data, 17 Registries, November 2021 sub (2000–2019) as the data source. It was a retrospective cohort study conducted in compliance with the Declaration of Helsinki. Ethically, informed consent was not required since the SEER database is publicly available and de-identified. This population-based cancer registry collects information on cancer incidence, prevalence, survival, and mortality in the United States, covering approximately 35% of the population. It includes data on patient demographics, tumor characteristics, treatment, and outcomes. 

For the study population, ovarian cancer patients diagnosed between 2000 and 2019 were identified using the International Classification of Diseases for Oncology, Third Edition (ICD-O-3) site code C56.9. Patients with missing data on key variables were excluded. The primary outcome variable was cardiovascular COD, encompassing heart disease, hypertension, cerebrovascular disease, peripheral vascular disease, aortic dissection, and other cardiovascular diseases. Age and race were among the exposure variables analyzed. 

Data exploration involved univariate and multivariate logistic regression analyses to identify risk factors for cardiovascular COD in ovarian cancer patients. Odds ratios (ORs) and 95% confidence intervals (CIs) were calculated for each exposure variable. Statistical significance was set at *p* < 0.05, and SPSS software was used for the analyses.

## 3. Results

### Baseline Characteristics

Our cohort data (*n* = 110,000 ovarian cancer patients) presents a summary of cancer cases and their causes of death, with a particular focus on cardiovascular causes. The data set includes 41,930 cases of patients who were alive, 54,829 cases of cancer-related deaths, 3003 cases of cardiovascular-related deaths, and 10,238 cases with other causes of death. Table 1 summarizes the baseline characteristics of our cohort.

Most of the patients were white (80.2%), followed by other patients (11.9%) and black patients (6.9%). The year of diagnosis ranged from 2000 to 2019, with the majority of cases diagnosed between 2011 and 2019 (61.7%). The laterality of the cancer was predominantly unilateral (70.47%), and 29.53% of cases had bilateral involvement. The staging of cancer was categorized into un-staged, regional, localized, and distant. The highest percentage of cancer cases had distant metastasis (23.91%), followed by localized (30.59%) and regional (9.02%) spread. Cancer cells were graded as follows: well-differentiated (grade I), moderately differentiated (grade II), poorly differentiated (grade III), and undifferentiated or anaplastic (grade IV). The majority of patients (19.83%) had poorly differentiated cancer cells (Grade III).

Regarding the cause of death, cardiovascular causes accounted for 3003 deaths (5.48%), with the majority of cases being in white people (84.2%). The highest percentage of cardiovascular deaths occurred in patients diagnosed between 2000 and 2010 (74.76%). Distant metastasis was the most common staging among patients with cardiovascular death (45.8%), followed by localized (23.3%) and regional (9.9%). Poorly differentiated cancer cells (grade III) were the most common among patients with cardiovascular death (24.5%).

All in all, the data highlight the significant impact of cancer-related cardiovascular death among cancer patients, with poorly differentiated cancer cells and distant metastasis being associated with a higher risk of cardiovascular mortality. These findings emphasize the importance of monitoring and managing cardiovascular risk factors in cancer patients to reduce the likelihood of cardiovascular death. 

Table 2 shows the results of a logistic regression analysis conducted to identify the risk factors for cardiovascular COD in ovarian cancer patients. The analysis included both univariate and multivariate models.

In the univariate model, age, the year of diagnosis, race, laterality, first malignancy, and staging were all found to be significant risk factors for cardiovascular COD. The ORs and 95% CIs are presented for each risk factor in Table 2.

The multivariate model included all the significant risk factors identified in the univariate model. After adjusting for confounding factors, age, the year of diagnosis, race, laterality, and staging remained significant risk factors for cardiovascular COD. The ORs and 95% CIs for the multivariate model are also presented in Table 2.

All in all, the findings indicate a notable decrease in the risk of cardiovascular-related mortality among patients aged 31–60 compared to those aged ≤30 years. Conversely, individuals over 60 years old face a higher risk. Moreover, the risk of cardiovascular-related death rises with a later diagnosis year. Non-white patients are also more susceptible to cardiovascular mortality. Additionally, bilateral ovarian cancer, as opposed to unilateral, is linked to an elevated risk.

Overall, these findings suggest that age, the year of diagnosis, race, laterality, and staging are important factors to consider in the management and treatment of ovarian cancer patients to reduce the risk of cardiovascular cause of death.

## 4. Discussion

Ovarian cancer is a highly aggressive malignancy that often goes undetected until it reaches an advanced stage, making it a difficult disease to treat. While there have been improvements in the management and treatment of ovarian cancer, it remains a leading cause of cancer-related deaths among women. Unfortunately, ovarian cancer patients are also at an increased risk of developing cardiovascular disease, which is a leading cause of mortality worldwide [2,6,7,8].

This study aimed to the identify risk factors associated with cardiovascular COD in ovarian cancer patients. The cohort consisted of 110,000 patients, and the study found that age, the year of diagnosis, race, laterality, and staging were all significant risk factors for cardiovascular death in ovarian cancer patients.

Cardiovascular comorbidity and chronic stress may affect the prognosis of ovarian cancer, which remains unfavorable despite therapeutic advancements. A retrospective cohort study using prospectively collected data discovered that CVD and stress-related markers are linked to survival rates among ovarian cancer patients. The effective management of specific cardiovascular comorbidities could enhance survival outcomes in these patients [9].

Another study found that cancer patients are at higher risk of dying from heart disease, with cancer survivors with certain types of cancer being about as likely to die of cardiovascular diseases as they are to die of their initial cancer [10]. Several studies also found that the risk of death from cardiovascular diseases is several times higher in cancer survivors than in the general population [9,10]. An investigation examining the association between pre-diagnosis cardiovascular health and total and cause-specific mortality among postmenopausal women who developed local- or regional-stage invasive cancer found that cardiometabolic abnormalities are a leading cause of death among women, including women with cancer [11]. This study found that most participants had one or two cardiometabolic risk factors, and the careful management of these risk factors may improve survival in cancer patients [11]. A review article found that cardiovascular disease and cancer share many risk factors, including age, smoking, physical inactivity, unhealthy diet, and obesity [12]. The article suggests that addressing these shared risk factors may help prevent both cardiovascular disease and cancer [12]. Overall, these studies suggest that the careful management of cardiovascular comorbidities and risk factors may improve survival in ovarian cancer patients and cancer patients in general.

The management of cardiovascular comorbidities in ovarian cancer patients may include lifestyle changes, such as exercise and a healthy diet, as well as medications to control blood pressure, cholesterol, and blood sugar levels. Additionally, managing cardiovascular comorbidities may involve treating and preventing complications, such as thrombosis, pericardial disease/tamponade, chronic obstructive pulmonary disease/pulmonary hypertension, and venous thromboembolism (VTE) [4]. For example, in a study by Hanley et al., the relationship between statin and β-blocker use and survival rates was examined in individuals diagnosed with epithelial ovarian cancer in British Columbia, Canada, between 1997 and 2015. The study addressed previous research limitations by using time-dependent analyses, avoiding immortal time bias and limited power. The findings showed that statin use after diagnosis was associated with improved survival rates for ovarian cancer, both in the overall cohort and among women with serious cancers. These results suggest that statins might have the potential to improve the prognosis of ovarian cancer patients. However, further research is needed to confirm these findings [13]. 

Cardiac biomarkers hold promise for assessing risk, predicting treatment response, diagnosing cardiotoxicity, monitoring disease progression, and evaluating the prognosis of cancer-related heart issues. However, their role prior to chemotherapy remains largely unknown. Pre-chemotherapy measurements of cardiac biomarkers can predict cardiotoxicity risk and identify patients who may benefit from cardioprotective therapies. Nonetheless, several challenges remain, including pre-existing cardiovascular disease and the lack of standardized protocols for measuring cardiac biomarkers. Further research is needed to explore their potential use in pre-chemotherapy risk assessment and management [14,15]. 

The early detection of cancer plays a vital role in the effective treatment and survival of patients. Biomarkers are objective characteristics utilized to achieve this goal. A reliable biomarker should possess diagnostic qualities, enable early detection, offer prognostic insights, and confirm treatment efficacy. Enzymes, metabolites, DNA/RNA, and surface receptors are among the various types of cancer biomarkers. Well-known examples include the prostate-specific antigen and carcinoembryonic antigen. Recent studies have focused on identifying new biomarkers that intersect between malignancy and cardiovascular disease. This research aims to enhance our understanding of the complex relationship between these two conditions. Patients affected by both cardiovascular disease and cancer face a higher mortality rate than those with either condition alone, underscoring the importance of addressing both diseases concurrently. The identification and investigation of shared biomarkers hold promise for diagnostic and prognostic purposes [16,17,18]. 

Our multivariate regression yielded non-significant results concerning the association between performing surgery and cardiovascular causes of death. This lack of significance could be attributed to the potential influence of radiotherapy and chemotherapy, which might have a substantial impact on cardiovascular mortality. Unfortunately, we were unable to investigate these factors further due to the unavailability of relevant data.

While our regression analysis did not yield significant results, it is important to acknowledge that certain surgeries or patient positions, such as the Trendelenburg position, can have significant effects on the cardiovascular system [19]. Studies have shown that even a 30 min increase in the duration of surgery for patients with epithelial ovarian cancer resulted in a notable increase in cardiovascular events and respiratory events [20]. These findings highlight the importance of considering cardiovascular risks associated with specific surgical techniques and patient positions, and further research in this area is needed to improve patient outcomes.

There are some limitations to our study that should be acknowledged. Firstly, the SEER database is limited to cancer cases within the United States and may not be representative of other populations or regions. Additionally, the database only includes cases of patients who received a cancer diagnosis and may not account for cases in which ovarian cancer went undiagnosed. Furthermore, our study did not consider potential lifestyle and environmental factors that could have influenced cardiovascular mortality in ovarian cancer patients. Lastly, due to an unavailability of data, we were not able to investigate the impact of radiotherapy and biological agents/chemotherapy on the cardiovascular mortality.

To address these limitations, future research should consider larger and more diverse populations, including international data, to better understand the prevalence and risk factors associated with cardiovascular mortality in ovarian cancer patients. Additionally, future studies could investigate the impact of lifestyle factors, such as physical activity and diet, on cardiovascular outcomes in ovarian cancer patients. Further research could also focus on developing personalized strategies for monitoring and managing cardiovascular risk factors in ovarian cancer patients.

## 5. Conclusions

In conclusion, our study highlights the need for increased awareness and management of cardiovascular risk factors in ovarian cancer patients in order to reduce the likelihood of cardiovascular death. While our findings provide insight into the characteristics of ovarian cancer patients and their causes of death, future research is necessary to address the limitations of our study and develop more personalized and effective approaches to managing cardiovascular risk in this population.

## Figures and Tables

**Table 1:** The baseline characteristics of our cohort.

	Alive (*n* = 41,930)	Cancer-Related Death (*n* = 54,829)	Cardiovascular-Related Death (*n* = 3003)	Other Causes of Death (*n* = 10,238)
**Race**				
White	80.2% (33,650)	84.6% (46,394)	84.2% (2529)	82.8% (8480)
Black	6.9% (2903)	8.4% (4577)	9.4% (282)	9.8% (1006)
Others	11.9% (4981)	6.9% (3796)	6.4% (192)	7.17% (734)
**Year of Diagnosis**				
2000–2010	38.4% (16,079)	65.2% (35,769)	74.76% (2245)	65.84% (6741)
2011–2019	61.7% (25,851)	34.8% (19,060)	25.4% (763)	34.16% (3497)
**Laterality**				
Unilateral	70.47% (29,549)	36.6% (20,080)	56.68% (1702)	50.87% (5208)
Bilateral	29.53% (12,381)	63.4% (34,749)	43.5% (1306)	49.13% (5030)
**Staging**				
Un-staged	2.77% (1160)	8.7% (4753)	13.82% (415)	9.7% (995)
Regional	9.02% (3784)	5.2% (2849)	9.9% (296)	8.7% (892)
Localized	30.59% (12,827)	3.6% (1960)	23.3% (699)	16.8% (1724)
Distant	23.91% (10,024)	72.2% (39,605)	45.8% (1375)	54.1% (5541)
**Grading**				
Well differentiated; grade I	11.86% (4971)	1.9% (1018)	6.8% (203)	5.8% (598)
Moderately differentiated; grade II	13.84% (5802)	7.9% (4308)	11.9% (358)	10.8% (1108)
Poorly differentiated; grade III	19.83% (8313)	30.5% (16,723)	24.5% (736)	24.5% (2503)
Undifferentiated; anaplastic; grade IV	11.63% (4878)	13.3% (7282)	8.4% (251)	9.7% (993)

Data are presented as % and numbers (*n*).

**Table 2:** Logistic Regression Model for analyzing the risk factors for cardiovascular cause of death in ovarian cancer patients.

Subject Characteristics	Univariate				Multivariate			
	OR	95% CI (LL)	95% CI (UL)	*p*-Value	OR	95% CI (LL)	95% CI (UL)	*p*-Value
Age								
≤30	Reference				Reference			
31–60	0.01	0.003	0.049	<0.001	0.008	0.002	0.03	<0.001
>60	0.27	0.23	0.28	<0.001	0.21	0.19	0.23	<0.001
Year of diagnosis								
2000–2005	Reference				Reference			
2006–2010	4.28	3.71	4.94	<0.001	2.38	2.15	2.63	<0.001
2011–2015	2.82	2.43	3.27	<0.001	1.54	1.38	1.72	<0.001
2015–2019	1.85	1.58	2.16	<0.001				
Race								
White	Reference				Reference			
Black	1.42	1.22	1.64	<0.001	1.06	0.91	1.24	0.46
Others	1.65	1.37	1.98	<0.001	1.37	1.13	1.68	<0.001
Laterality								
Unilateral	Reference				Reference			
Bilateral	1.24	1.15	1.33	<0.001	1.37	1.25	1.49	<0.001
First Malignancy	1.23	1.12	1.36	<0.001	1.06	0.96	1.18	0.25
Staging								
Un-staged	Reference				Reference			
Localized	1.59	1.39	1.80	<0.001	1.84	1.61	2.10	<0.001
Regional	2.41	2.15	2.70	<0.001	2.20	1.96	2.47	<0.001
Distant	1.70	1.55	1.86	<0.001	2.43	2.19	2.70	<0.001
Surgery performed	0.596	0.552	0.642	<0.001	0.981	0.896	1.074	0.682

CI: confidence interval; LL: lower limit; OR: odds ratio; UL: upper limit.

## Data Availability

Data was obtained from a publicly available database under the Hospital Cost and Utilization Project’s Nationwide Inpatient Sample, which can be accessed: https://www.hcup-us.ahrq.gov/db/nation/nis/nisdbdocumentation.jsp.
